# Inferences for Exponentiated Gamma Constant-Stress Partially Accelerated Life Test Model Based on Generalized Type-I Hybrid Censored Data

**DOI:** 10.1155/2021/5424630

**Published:** 2021-12-27

**Authors:** Abdalla Rabie, Abd-EL-Baset A. Ahmad, Thierno Souleymane Barry, Hassan M. Aljohani, Nada M. Alfaer, Abdulaziz S. Alghamdi

**Affiliations:** ^1^Department of Mathematics, Faculty of Science, Al-Azhar University, Assiut 71524, Egypt; ^2^Department of Mathematics, Faculty of Science, Assiut University, Assiut, Egypt; ^3^Mathematics (Statistics Option) Program, Pan African University, Institute of Basic Sciences, Technology and Innovation (PAUISTI), P. O. BOX 62000-00200, Nairobi, Kenya; ^4^Department of Mathematics & Statistics, College of Science, Taif University, P. O. Box 11099, Taif 21944, Saudi Arabia; ^5^Department of Mathematics, College of Science & Arts, King Abdulaziz University, P. O. Box 344, Rabigh 21911, Saudi Arabia

## Abstract

In this paper, the exponentiated gamma distribution (EGD) with generalized Type-I hybrid censored data under constant-stress partially accelerated life test (CSPALT) model is considered. The Bayesian and E-Bayesian estimation methods, as well as the maximum likelihood estimation method, are discussed for the parameter of the distribution and the acceleration factor. The E-Bayesian and Bayesian estimates are derived by using the squared error loss (SEL) and the LINEX loss functions. The MCMC method is applied for deriving the Bayesian and then E-Bayesian estimates. Moreover, a real data set is given for the illustrative purpose. After all, an evaluation is performed for the results of the proposed methods.

## 1. Introduction

Many types of censoring schemes are used in the last decades. Type-I and Type-II censoring methods are still the most often used censoring schemes. In Type-I censoring, the test is terminated at a specified time *τ* and the number of failures is random. In Type-II censoring, the test is ended after obtaining a prefixed number of failures while the time of the test is random. In these two types, the experimenter does not know when he will finish the test (as in Type-II) or he can get the required number before time *τ* (as in Type-I). To overcome these disadvantages of Type-I and Type-II, Epstein [1] introduced a mixture of Type-I and Type-II and referred to as hybrid censoring scheme (HCS) mainly, Type-I HCS and Type-II HCS. In Type-I HCS, the test is terminated at a random time *T*_*∗*_ = min{*X*_*r*:*n*_, *T*}, where *r* ∈ {1,2,…, *n*} and *T* ∈ (0, *∞*) are fixed from the begining of the test. Regarding Type-II HCS, the examination is terminated at a random point of time, and let us say it *T*^*∗*^ = max{*X*_*r*:*n*_, *T*}. Also, these schemes have drawbacks like having a few number of failures or not knowing the maximum time to finish the test. Therefore, Chandrasekar et al. [2] proposed an efficient and new censoring scheme which known as the generalized hybrid censoring schemes (generalized HCSs) to overcome drawbacks of HCSs. These schemes are considered as an extension of Type-I and Type-II HCSs. Therefore, we can notice that two types of censoring schemes are defined along these lines.

Generalized Type-I HCS: let *k*, *r* ∈ {1,2,…, *n*} be integers and *k* < *r* < *n*, with time point *T* ∈ (0, *∞*). In this scheme, the test is ended at min{*X*_*r*:*n*_, *T*} when the *k*-th failure happens before *T*. If the *k*-th failure is occurred next to *T*, the experiment is terminated at *X*_*k*:*n*_. Generalized Type-II HCS: set *r* ∈ {1,2,…, *n*} with time points *T*_1_, *T*_2_ ∈ (0, *∞*) where *T*_1_ < *T*_2_. If the *r*-th failure occurs sooner than time *T*_1_, the experiment is finished at *T*_1_. If the *r*-th failure is obtained between *T*_1_ and *T*_2_, the experiment is terminated at *X*_*r*:*n*_. In the end, if the *r*-th failure occurs after time *T*_2_, the test is terminated at *T*_2_. These schemes are studied by many authors such as Huang and Yang [3]; Rabie, and Li [4]; and Rabie and Li [5].

Due to the continuous enhancement in industrial design, it is difficult to get enough information of the products in the reliability tests under normal use conditions. Therefore, the accelerated life test (ALT) and partially accelerated life test (PALT) are used for this purpose. In these tests, a sample is subjected to more severe operating conditions than normal use conditions to obtain rapid failures. ALT items are allocated only in accelerated condition, while PALT is applied with normal and accelerated use conditions. This technique of tests results in shorter lifetimes than under normal use conditions. Extreme stress can be applied in several ways; the most common methods are step-stress and constant-stress as designated in Nelson [6]. In step-stress PALT, units first run at normal use condition; if units do not fail at a specified time, they allocated at accelerated use condition. Under constant stress, test items are divided into two groups: one of them runs at normal use conditions and the other is subjected to accelerated use conditions. We focus on the CSPALT model with generalized Type-I HCS in this paper.

### 1.1. The Model Description and Test Procedures

The exponentiated gamma distribution (EGD) was suggested by Gupta et al. [7] as an alternative to Weibull and gamma distributions. This study suggested that the EGD can present a better fit to the real data set than the GD. The cumulative distribution function (CDF), probability density function (PDF), and the reliability function (R(t)) of the EGD are written, respectively, in the forms as follows:

(1) $$\begin{array}{c} F\left(x;\theta \right)={\left(1-e^{-x}\left(x+1\right)\right)}^{\theta },\;x>0,\left(\theta >0\right), \end{array}$$

(2) $$\begin{array}{c} f\left(x;\theta \right)=\theta xe^{-x}{\left(1-e^{-x}\left(x+1\right)\right)}^{\theta -1},\;x>0,\left(\theta >0\right), \end{array}$$

(3) $$\begin{array}{c} R\left(x;\theta \right)=1-{\left(1-e^{-x}\left(x+1\right)\right)}^{\theta },\;x>0,\left(\theta >0\right), \end{array}$$

where *θ* is the shape parameter. It is noted that when *θ*=1, the EGD turns into *G*(2,1); for more details, one can refer to Shawky and Bakoban [8–10], Singh et al. [11]; Khan and Kumar [12]; Ghanizadeh et al. [13]; and Feroze and Aslam [14].

**Figure 1:**
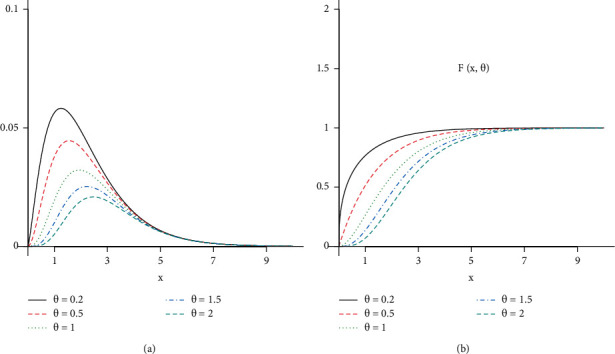
Plots the shapes of the PDF (a) and CDF (b) of the EGD.

Figure 1 shows the plots of the shape of the PDF and CDF of the EGD distribution; it can be seen that the PDF has a unique mode as the parameter *θ* decreases. The distance between the shape of CDF increases as the parameter *θ* increases.

In CSPALT, a sample of size *n* of test items is divided into two groups *n*_1_ and *n*_2_ chosen randomly among *n* items. *n*_1_ items run at normal use conditions, while *n*_2_ items are allocated at accelerated use conditions at the same time. The experiment is planned to continue at most until time *T* in both normal use and accelerated use conditions. We desire to obtain *r*_1_and *r*_2_ failures out of *n*_1_ and *n*_2_, respectively. And a bare minimum acceptable number of failures are *k*_1_ and *k*_2_ from *n*_1_ and *n*_2_, respectively. According to the CSPALT model under generalized Type-I HCS, one can note the following three cases of censored data. For normal use conditions, we observe

  Case I: {*X*_1:*n*_1__ < *X*_2:*n*_1__ < ⋯<*X*_*k*_1_:*n*_1__} if *X*_*k*_1_:*n*_1__ > *T*
  Case II: {*X*_1:*n*_1__ < ⋯<*X*_*k*_1_:*n*_1__ < ⋯<*X*_*r*_1_:*n*_1__} if *X*_*r*_1_:*n*_1__ < *T*
  Case III: {*X*_1:*n*_1__ < ⋯<*X*_*k*_1_:*n*_1__ < ⋯<*X*_*d*_1_:*n*_1__} if *X*_*r*_1_:*n*_1__ > *T*

where *d*_1_ denotes the number of failures occurring up to time *T* in the case of normal use conditions.

Also, for accelerated use conditions, we observe the following three cases:

  Case 1: {*Y*_1:*n*_2__ < *Y*_2:*n*_2__ < ⋯<*Y*_*k*_1_:*n*_2__} if *Y*_*k*_2_:*n*_2__ > *T*
  Case 2: {*Y*_1:*n*_2__ < ⋯<*Y*_*k*_2_:*n*_2__ < ⋯<*Y*_*r*_2_:*n*_2__} if *Y*_*r*_2_:*n*_2__ < *T*
  Case 3: {*Y*_1:*n*_2__ < ⋯<*Y*_*k*_2_:*n*_2__ < ⋯<*Y*_*d*_2_:*n*_2__} if *Y*_*r*_2_:*n*_2__ > *T*

where *Y*=*λ*^−1^*X*, *λ* is the acceleration factor, and *d*_2_ stands for the number of failures occurring up to *T* in the case of accelerated use conditions.

The remainder of this article is organised as follows: In Section 2, the maximum likelihood based on CSPALT generalized Type-I HCS is discussed. In Section 3, the Bayesian estimation is studied under the SEL and LINEX loss functions by using the MCMC method. In Section 4, we present the Bayesian estimates based on the MCMC method. In Section 5, we present the E-Bayesian estimates based on the SEL and LINEX loss functions. In Section 6, we present the simulation study of the algorithm. We examine the flexibility of the distribution to fit the accelerated data in Section 7, so we provided a real data example, and the numerical results concluded are presented to asses the performance of the distribution.

## 2. The Likelihood Function Based on Constant-Stress Generalized Type-I Hybrid Censoring Scheme

We assume that *X*_1_, *X*_2_,…, *X*_*n*_1__ are *n*_1_ observations of failure lifetimes under typical usage conditions that follows the generalized Type-I HCS, and *Y*_1_, *Y*_2_,…, *Y*_*n*_2__ are *n*_2_ observations of breakdown lifetimes under accelerated usage conditions that follows the generalized Type-I HCS. The lifespan of test items is determined by EGD. So we can refer to the PDF under typical usage situation as in equation (2), and the following PDF is presented for a product in an accelerated consumption stage which can be written as the following:

(4) $$\begin{array}{c} f\left(y;\theta ,\lambda \right)=\theta \lambda^{2}ye^{-\lambda y}{\left(1-e^{-\lambda y}\left(\lambda y+1\right)\right)}^{\theta -1},\;y,\theta >0,\;\lambda >1, \end{array}$$

where *Y* = *λ*^−1^*X*. Therefore, if *d*_1_ denotes failures number obtained before *T*, so we can write the likelihood equation under generalized Type-I HCS for (*x*_*j*_; *θ*), *j* = 1,2,…, *n*_1_, without the multiplicative constant in usual usage is provided as

(5) $$\begin{array}{c} L\left(\theta \right)=\left\{\begin{array}{c} \theta^{k_{1}}{\left\{1-\varphi_{k_{1}}^{\theta }\right\}}^{n_{1}-k_{1}}\prod_{i=1}^{k_{1}}x_{i}e^{-x_{i}}\varphi_{i}^{\theta -1},\\ d_{1}=0,1,\ldots ,k_{1}-1,\\ \theta^{d_{1}}{\left\{1-\varphi_{T}^{\theta }\right\}}^{n_{1}-d_{1}}\prod_{i=1}^{d_{1}}x_{i}e^{-x_{i}}\varphi_{i}^{\theta -1},\\ d_{1}=k_{1},k_{1}+1,\ldots ,r_{1}-1,\\ \theta^{r_{1}}{\left\{1-\varphi_{r_{1}}^{\theta }\right\}}^{n_{1}-r_{1}}\prod_{i=1}^{r_{1}}x_{i}e^{-x_{i}}\varphi_{i}^{\theta -1},\\ d_{1}=r_{1}, \end{array}\right. \end{array}$$

where *φ*_*t*_ = 1 − *e*^−*t*^(*t* + 1), and for (*y*_*j*_; *θ*, *λ*); *j* = 1,2,…, *n*_2_, if *d*_2_ indicates the number of failures that occur prior to the specified period *T*, we can refer to the likelihood equations without the multiplicative constant in accelerated usage which is provided as

(6) $$\begin{array}{c} L\left(\theta ,\lambda \right)=\left\{\begin{array}{c} \theta^{k_{2}}\lambda^{2k_{2}}{\left\{1-\psi_{k_{2}}^{\theta }\right\}}^{n_{2}-k_{2}}\prod_{j=1}^{k_{2}}y_{j}e^{-\lambda y_{j}}\psi_{j}^{\theta -1},\\ d_{2}=0,1,\ldots ,k_{2}-1,\\ \theta^{d_{2}}\lambda^{2d_{2}}{\left\{1-\psi_{T}^{\theta }\right\}}^{n_{2}-d_{2}}\prod_{j=1}^{d_{2}}y_{j}e^{-\lambda y_{j}}\psi_{j}^{\theta -1},\\ d_{2}=k_{2},k_{2}+1,\ldots ,r_{2}-1,\\ \theta^{r_{2}}\lambda^{2r_{2}}{\left\{1-\psi_{r_{2}}^{\theta }\right\}}^{n_{2}-r_{2}}\prod_{j=1}^{r_{2}}y_{j}e^{-\lambda y_{j}}\psi_{j}^{\theta -1},\\ d_{2}=r_{2}, \end{array}\right. \end{array}$$

where *ψ*_*t*_ = 1 − *e*^−*λt*^(*λt* + 1). By combining equations (5) and (6), the total likelihood function for {(*x*_*i*_; *θ*), (*y*_*j*_; *θ*, *λ*) :  *i* = 1,…, *n*_1_;  *j* = 1,…, *n*_2_} can be written as follows:

(7) $$\begin{array}{c} L\left(\theta ,\lambda \right)=\left\{\begin{array}{c} \theta^{k_{1}+k_{2}}\lambda^{2k_{2}}{\left\{1-\varphi_{k_{1}}^{\theta }\right\}}^{n_{1}-k_{1}}{\left\{1-\psi_{k_{2}}^{\theta }\right\}}^{n_{2}-k_{2}}\prod_{i=1}^{k_{1}}x_{i}e^{-x_{i}}\varphi_{i}^{\theta -1}\\ \times \prod_{j=1}^{k_{2}}y_{j}e^{-\lambda y_{j}}\psi_{j}^{\theta -1},d_{1}=0,1,\ldots ,k_{1}-1,d_{2}=0,1,\ldots ,k_{2}-1,\\ \theta^{d_{1}+d_{2}}\lambda^{2d_{2}}{\left\{1-\varphi_{T}^{\theta }\right\}}^{n_{1}-d_{1}}{\left\{1-\psi_{T}^{\theta }\right\}}^{n_{2}-d_{2}}\prod_{i=1}^{d_{1}}x_{i}e^{-x_{i}}\varphi_{i}^{\theta -1}\\ \times \prod_{j=1}^{d_{2}}y_{j}e^{-\lambda y_{j}}\psi_{j}^{\theta -1},d_{1}=k_{1},k_{1}+1,\ldots ,r_{1}-1,d_{2}=k_{2},k_{2}+1,\ldots ,r_{2}-1,\\ \theta^{r_{1}+r_{2}}\lambda^{2r_{2}}{\left\{1-\varphi_{r_{1}}^{\theta }\right\}}^{n_{1}-r_{1}}{\left\{1-\psi_{r_{2}}^{\theta }\right\}}^{n_{2}-r_{2}}\prod_{i=1}^{r_{1}}x_{i}e^{-x_{i}}\varphi_{i}^{\theta -1}\\ \times \prod_{j=1}^{r_{2}}y_{j}e^{-\lambda y_{j}}\psi_{j}^{\theta -1},d_{1}=r_{1},d_{2}=r_{2}. \end{array}\right.\; \end{array}$$

### 2.1. Maximum Likelihood Estimation Method

As it is stated, the log-likelihood is monotonically increasing so maximizing the likelihood function is equivalent to maximizing the log-likelihood. By calculating the log of the expression (7), as proceeds, we obtain the log-likelihood function:

(8) $$\begin{array}{c} \ell =\ln\;L\left(\theta ,\lambda \right)\\ =\left\{\begin{array}{c} \left(k_{1}+k_{2}\right)\ln\;\theta +2k_{2}\ln\;\lambda +\left(n_{1}-k_{1}\right)\ln\left\{1-\varphi_{k_{1}}^{\theta }\right\}+\left(n_{2}-k_{2}\right)\ln\left\{1-\psi_{k_{2}}^{\theta }\right\}\\ +\sum_{i=1}^{k_{1}}\ln x_{i}-\sum_{i=1}^{k_{1}}x_{i}+\left(\theta -1\right)\sum_{i=1}^{k_{1}}\ln\left(\varphi_{i}\right)+\sum_{j=1}^{k_{2}}\ln y_{j}-\sum_{j=1}^{k_{2}}\lambda y_{j}+\left(\theta -1\right)\sum_{j=1}^{k_{2}}\ln\left(\psi_{j}\right),\\ d_{1}=0,1,\ldots ,k_{1}-1,d_{2}=0,1,\ldots ,k_{2}-1,\\ \left(d_{1}+d_{2}\right)\ln\;\theta +2d_{2}\ln\;\lambda +\left(n_{1}-d_{1}\right)\ln\left\{1-\varphi_{T}^{\theta }\right\}+\left(n_{2}-d_{2}\right)\ln\left\{1-\psi_{T}^{\theta }\right\}\\ +\sum_{i=1}^{d_{1}}\ln x_{i}-\sum_{i=1}^{d_{1}}x_{i}+\left(\theta -1\right)\sum_{i=1}^{d_{1}}\ln\left(\varphi_{i}\right)+\sum_{j=1}^{d_{2}}\ln y_{j}-\lambda \sum_{j=1}^{d_{2}}y_{j}+\left(\theta -1\right)\sum_{j=1}^{d_{2}}\ln\left(\psi_{j}\right),\\ d_{1}=k_{1},k_{1}+1,\ldots ,r_{1}-1,d_{2}=k_{2},k_{2}+1,\ldots ,r_{2}-1,\\ \left(r_{1}+r_{2}\right)\ln\;\theta +2r_{2}\ln\;\lambda +\left(n_{1}-r_{1}\right)\ln\left\{1-\varphi_{r_{1}}^{\theta }\right\}+\left(n_{2}-r_{2}\right)\ln\left\{1-\psi_{r_{2}}^{\theta }\right\}\\ +\sum_{i=1}^{r_{1}}\ln x_{i}-\sum_{i=1}^{r_{1}}x_{i}+\left(\theta -1\right)\sum_{i=1}^{r_{1}}\ln\left(\varphi_{i}\right)+\sum_{j=1}^{r_{2}}\ln y_{j}-\lambda \sum_{j=1}^{r_{2}}y_{j}+\left(\theta -1\right)\sum_{j=1}^{r_{2}}\ln\left(\psi_{j}\right),\\ d_{1}=r_{1},d_{2}=r_{2}. \end{array}\right. \end{array}$$

Set the first derivatives of equation (8) regarding *θ* and *λ* to zero and solve the following equations numerically:

(9) $$\begin{array}{c} \frac{\partial \ell }{\partial \theta }=0,\\ \frac{\partial \ell }{\partial \lambda }=0, \end{array}$$

to get maximum likelihood estimates (MLEs) of *θ* and *λ*.

## 3. Bayesian Analysis

In this part of the paper, we made the most important estimation technique which is the Bayesian estimation for the two parametres *θ* and *λ*. We made estimation using diffirent estimation loss functions such as the SEL and LINEX loss functions. We assumed that the test follows CSPALT generalized Type-I hybrid censored sample from EGD. We uesd gamma prior for the two parameters *θ* and *λ* like gamma (*a*_1_, *a*_2_) and gamma (*b*_1_, *b*_2_), respectively. So, we can write the joint prior PDF equation of the two parameters of the distribution *θ* and *λ*, and it will have the following form:

(10) $$\begin{array}{c} \pi \left(\theta ,\lambda \right)=\pi_{1}\left(\theta \right)\pi_{2}\left(\lambda \right)\\ \propto \theta^{a_{1}-1}\lambda^{b_{1}-1}e^{-\left(a_{2}\theta +b_{2}\lambda \right)}, \end{array}$$

where

(11) $$\begin{array}{c} \pi_{1}\left(\theta \right)\propto \theta^{a_{1}-1}\exp\left(-a_{2}\theta \right),\;\left(a_{1},a_{2}>0\right),\theta >0,\\ \pi_{2}\left(\lambda \right)\propto \lambda^{b_{1}-1}\exp\left(-b_{2}\lambda \right),\;\left(b_{1},b_{2}>0\right),\lambda >1. \end{array}$$

Then, the joint posterior PDF of *θ* and *λ* is written from (7) and (10) as follows:

(12) $$\begin{array}{c} \pi^{*}\left(\theta ,\lambda |x_{i},y_{j}\right)=\left\{\begin{array}{c} \frac{1}{K}\theta^{k_{1}+k_{2}+a_{1}-1}\lambda^{2k_{2}+b_{1}-1}{\left\{1-\varphi_{k_{1}}^{\theta }\right\}}^{n_{1}-k_{1}}{\left\{1-\psi_{k_{2}}^{\theta }\right\}}^{n_{2}-k_{2}}\prod_{i=1}^{k_{1}}x_{i}e^{-x_{i}}\varphi_{i}^{\theta -1}\\ \times e^{-\left(a_{2}\theta +b_{2}\lambda \right)}\prod_{j=1}^{k_{2}}y_{j}e^{-\lambda y_{j}}\psi_{j}^{\theta -1},d_{1}=0,1,\ldots ,k_{1}-1,d_{2}=0,1,\ldots ,k_{2}-1,\\ \frac{1}{K}\theta^{d_{1}+d_{2}+a_{1}-1}\lambda^{2d_{2}+b_{1}-1}{\left\{1-\varphi_{T}^{\theta }\right\}}^{n_{1}-d_{1}}{\left\{1-\psi_{T}^{\theta }\right\}}^{n_{2}-d_{2}}\prod_{i=1}^{d_{1}}x_{i}e^{-x_{i}}\varphi_{i}^{\theta -1}\\ \times e^{-\left(a_{2}\theta +b_{2}\lambda \right)}\prod_{j=1}^{d_{2}}y_{j}e^{-\lambda y_{j}}\psi_{j}^{\theta -1},d_{1}=k_{1},\ldots ,r_{1}-1,d_{2}=k_{2},\ldots ,r_{2}-1,\\ \frac{1}{K}\theta^{r_{1}+r_{2}+a_{1}-1}\lambda^{2r_{2}+b_{1}-1}{\left\{1-\varphi_{r_{1}}^{\theta }\right\}}^{n_{1}-r_{1}}{\left\{1-\psi_{r_{2}}^{\theta }\right\}}^{n_{2}-r_{2}}\prod_{i=1}^{r_{1}}x_{i}e^{-x_{i}}\varphi_{i}^{\theta -1}\\ \times e^{-\left(a_{2}\theta +b_{2}\lambda \right)}\prod_{j=1}^{r_{2}}y_{j}e^{-\lambda y_{j}}\psi_{j}^{\theta -1},d_{1}=r_{1},d_{2}=r_{2}, \end{array}\right. \end{array}$$

where  *K* is a normalizing constant defined by

(13) $$\begin{array}{c} K=\int_{1}^{\infty }\int_{0}^{\infty }\pi^{*}\left(\theta ,\lambda |x_{i},y_{j}\right)\text{d}\theta \text{d}\lambda . \end{array}$$

Under the SEL function, the Bayesian estimate of any function *u*(*θ*, *λ*) of *θ* and *λ* is given by

(14) $$\begin{array}{c} {\hat{u}}_{BS}=E\left[u\left(\theta ,\lambda \right)|x,y\right]\\ =\int_{1}^{\infty }\int_{0}^{\infty }u\left(\theta ,\lambda \right)\pi^{*}\left(\theta ,\lambda |x_{i},y_{j}\right)\text{d}\theta \text{d}\lambda \\ =\left\{\begin{array}{c} \frac{1}{K}\int_{1}^{\infty }\int_{0}^{\infty }u\left(\theta ,\lambda \right)\theta^{k_{1}+k_{2}+a_{1}-1}\lambda^{2k_{2}+b_{1}-1}{\left\{1-\varphi_{k_{1}}^{\theta }\right\}}^{n_{1}-k_{1}}{\left\{1-\psi_{k_{2}}^{\theta }\right\}}^{n_{2}-k_{2}}\prod_{i=1}^{k_{1}}x_{i}e^{-x_{i}}\varphi_{i}^{\theta -1}\\ \times e^{-\left(a_{2}\theta +b_{2}\lambda \right)}\prod_{j=1}^{k_{2}}y_{j}e^{-\lambda y_{j}}\psi_{j}^{\theta -1}\text{d}\theta \text{d}\lambda ,d_{1}=0,1,\ldots ,k_{1}-1,d_{2}=0,1,\ldots ,k_{2}-1,\\ \frac{1}{K}\int_{1}^{\infty }\int_{0}^{\infty }u\left(\theta ,\lambda \right)\theta^{d_{1}+d_{2}+a_{1}-1}\lambda^{2d_{2}+b_{1}-1}{\left\{1-\varphi_{T}^{\theta }\right\}}^{n_{1}-d_{1}}{\left\{1-\psi_{T}^{\theta }\right\}}^{n_{2}-d_{2}}\prod_{i=1}^{d_{1}}x_{i}e^{-x_{i}}\varphi_{i}^{\theta -1}\\ \times e^{-\left(a_{2}\theta +b_{2}\lambda \right)}\prod_{j=1}^{d_{2}}y_{j}e^{-\lambda y_{j}}\psi_{j}^{\theta -1}\text{d}\theta \text{d}\lambda ,d_{1}=k_{1},\ldots ,r_{1}-1,d_{2}=k_{2},\ldots ,r_{2}-1,\\ \frac{1}{K}\int_{1}^{\infty }\int_{0}^{\infty }u\left(\theta ,\lambda \right)\theta^{r_{1}+r_{2}+a_{1}-1}\lambda^{2r_{2}+b_{1}-1}{\left\{1-\varphi_{r_{1}}^{\theta }\right\}}^{n_{1}-r_{1}}{\left\{1-\psi_{r_{2}}^{\theta }\right\}}^{n_{2}-r_{2}}\prod_{i=1}^{r_{1}}x_{i}e^{-x_{i}}\varphi_{i}^{\theta -1}\\ \times e^{-\left(a_{2}\theta +b_{2}\lambda \right)}\prod_{j=1}^{r_{2}}y_{j}e^{-\lambda y_{j}}\psi_{j}^{\theta -1}\text{d}\theta \text{d}\lambda ,d_{1}=r_{1},d_{2}=r_{2}. \end{array}\right. \end{array}$$

Based on the LINEX loss function, the Bayesian estimate of *u*(*θ*, *λ*) is given by

(15) $$\begin{array}{c} {\hat{u}}_{BL}=\frac{-1}{h}\text{ln}E\left[e^{\left(-hu\left(\theta ,\lambda \right)\right)}|x,y\right]\\ =\frac{-1}{h}\text{ln}\int_{1}^{\infty }\int_{0}^{\infty }e^{\left(-hu\left(\theta ,\lambda \right)\right)}\pi^{*}\left(\theta ,\lambda |x_{i},y_{j}\right)\text{d}\theta \text{d}\lambda \\ =\left\{\begin{array}{c} \frac{-1}{Kh}\text{ln}\int_{1}^{\infty }\int_{0}^{\infty }\theta^{k_{1}+k_{2}+a_{1}-1}\lambda^{2k_{2}+b_{1}-1}{\left\{1-\varphi_{k_{1}}^{\theta }\right\}}^{n_{1}-k_{1}}{\left\{1-\psi_{k_{2}}^{\theta }\right\}}^{n_{2}-k_{2}}\prod_{i=1}^{k_{1}}x_{i}e^{-x_{i}}\varphi_{i}^{\theta -1}\\ \times e^{-\left(hu\left(\theta ,\lambda \right)+a_{2}\theta +b_{2}\lambda \right)}\prod_{j=1}^{k_{2}}y_{j}e^{-\lambda y_{j}}\psi_{j}^{\theta -1}\text{d}\theta \text{d}\lambda ,d_{1}=0,1,\ldots ,k_{1}-1,d_{2}=0,1,\ldots ,k_{2}-1,\\ \frac{-1}{Kh}\text{ln}\int_{1}^{\infty }\int_{0}^{\infty }\theta^{d_{1}+d_{2}+a_{1}-1}\lambda^{2d_{2}+b_{1}-1}{\left\{1-\varphi_{T}^{\theta }\right\}}^{n_{1}-d_{1}}{\left\{1-\psi_{T}^{\theta }\right\}}^{n_{2}-d_{2}}\prod_{i=1}^{d_{1}}x_{i}e^{-x_{i}}\varphi_{i}^{\theta -1}\\ \times e^{-\left(hu\left(\theta ,\lambda \right)+a_{2}\theta +b_{2}\lambda \right)}\prod_{j=1}^{d_{2}}y_{j}e^{-\lambda y_{j}}\psi_{j}^{\theta -1}\text{d}\theta \text{d}\lambda ,d_{1}=k_{1},\ldots ,r_{1}-1,d_{2}=k_{2},\ldots ,r_{2}-1,\\ \frac{-1}{Kh}\text{ln}\int_{1}^{\infty }\int_{0}^{\infty }\theta^{r_{1}+r_{2}+a_{1}-1}\lambda^{2r_{2}+b_{1}-1}{\left\{1-\varphi_{r_{1}}^{\theta }\right\}}^{n_{1}-r_{1}}{\left\{1-\psi_{r_{2}}^{\theta }\right\}}^{n_{2}-r_{2}}\prod_{i=1}^{r_{1}}x_{i}e^{-x_{i}}\varphi_{i}^{\theta -1}\\ \times e^{-\left(hu\left(\theta ,\lambda \right)+a_{2}\theta +b_{2}\lambda \right)}\prod_{j=1}^{r_{2}}y_{j}e^{-\lambda y_{j}}\psi_{j}^{\theta -1}\text{d}\theta \text{d}\lambda ,d_{1}=r_{1},d_{2}=r_{2}. \end{array}\right. \end{array}$$

It is clear from (14) and (15) that Bayesian estimates of *θ* and *λ* cannot be directly calculated, so the MCMC method is used for this purpose.

## 4. Bayesian Estimates Using MCMC Method

Here, we present the MCMC technique to compute and find the Bayesian estimates of *θ* and *λ*. The conditional posterior PDF of the parameter *θ* and the acceleration factor *λ* is, respectively, written as follows:

(16) $$\begin{array}{c} \pi^{*}\left(\theta |\lambda ;x_{i},y_{j}\right)=\left\{\begin{array}{c} \theta^{k_{1}+k_{2}+a_{1}-1}{\left\{1-\varphi_{k_{1}}^{\theta }\right\}}^{n_{1}-k_{1}}{\left\{1-\psi_{k_{2}}^{\theta }\right\}}^{n_{2}-k_{2}}\prod_{i=1}^{k_{1}}\varphi_{i}^{\theta -1}\\ \times e^{-a_{2}\theta }\prod_{j=1}^{k_{2}}\psi_{j}^{\theta -1},d_{1}=0,1,\ldots ,k_{1}-1,d_{2}=0,1,\ldots ,k_{2}-1,\\ \theta^{d_{1}+d_{2}+a_{1}-1}{\left\{1-\varphi_{T}^{\theta }\right\}}^{n_{1}-d_{1}}{\left\{1-\psi_{T}^{\theta }\right\}}^{n_{2}-d_{2}}\prod_{i=1}^{d_{1}}\varphi_{i}^{\theta -1}\\ \times e^{-a_{2}\theta }\prod_{j=1}^{d_{2}}\psi_{j}^{\theta -1},d_{1}=k_{1},\ldots ,r_{1}-1,d_{2}=k_{2},\ldots ,r_{2}-1,\\ \theta^{r_{1}+r_{2}+a_{1}-1}{\left\{1-\varphi_{r_{1}}^{\theta }\right\}}^{n_{1}-r_{1}}{\left\{1-\psi_{r_{2}}^{\theta }\right\}}^{n_{2}-r_{2}}\prod_{i=1}^{r_{1}}\varphi_{i}^{\theta -1}\\ \times e^{-a_{2}\theta }\prod_{j=1}^{r_{2}}\psi_{j}^{\theta -1},d_{1}=r_{1},d_{2}=r_{2}, \end{array}\right. \end{array}$$

and

(17) $$\begin{array}{c} \pi^{*}\left(\lambda |\theta ;x_{i},y_{j}\right)=\left\{\begin{array}{c} \lambda^{2k_{2}+b_{1}-1}{\left\{1-\psi_{k_{2}}^{\theta }\right\}}^{n_{2}-k_{2}}e^{-b_{2}\lambda }\prod_{j=1}^{k_{2}}e^{-\lambda y_{j}}\psi_{j}^{\theta -1},\\ d_{1}=0,1,\ldots ,k_{1}-1,d_{2}=0,1,\ldots ,k_{2}-1,\\ \lambda^{2d_{2}+b_{1}-1}{\left\{1-\psi_{T}^{\theta }\right\}}^{n_{2}-d_{2}}e^{-b_{2}\lambda }\prod_{j=1}^{d_{2}}e^{-\lambda y_{j}}\psi_{j}^{\theta -1},\\ d_{1}=k_{1},\ldots ,r_{1}-1,d_{2}=k_{2},\ldots ,r_{2}-1,\\ \lambda^{2r_{2}+b_{1}-1}{\left\{1-\psi_{r_{2}}^{\theta }\right\}}^{n_{2}-r_{2}}e^{-\left(a_{2}\theta +b_{2}\lambda \right)}\prod_{j=1}^{r_{2}}e^{-\lambda y_{j}}\psi_{j}^{\theta -1},\\ d_{1}=r_{1},d_{2}=r_{2}. \end{array}\right.\; \end{array}$$

As it is seen from equations (16) and (17), the conditional posterior PDF of *θ* and *λ* does not look like any well-known models. Therefore, we use Metropolis-Hastings techinque to produce samples of *θ* and *λ* from the conditional posterior PDF using normal proposal distribution. Posterior samples of *θ* and *λ* are, respectively, generated from equations (16) and (17) using the Metropolis-Hastings algorithm.

Step 1: First we initiate with starting values of *θ* and *λ* and let it be the MLE values $\left({\hat{\theta }}_{MLE},{\hat{\lambda }}_{MLE}\right)$.
Step 2: take *j* = 1.
Step 3: from equation (16), generate *θ*^(*j*)^ and produce samples of *θ*^(*∗*)^ from a normal distribution as a proposal distribution.
Step 4: Now we will compute the probability of accepting or rejecting the generated sample which callled the acceptance probability by using the following equation:
  (18) $$\begin{array}{c} r\left(\theta^{j-1}|\theta^{\left(*\right)}\right)=\min\left[1,\frac{\pi^{*}\left(\theta^{\left(*\right)}|\lambda^{\left(j-1\right)}\right)}{\pi^{*}\left(\theta^{\left(j-1\right)}|\lambda^{\left(j-1\right)}\right)}\right]. \end{array}$$
Step 5: we will produce samples from the uniform distribution ranged from zero to one as the following:*U* ~ *U*(0,1).
Step 6: if *U* ≤ *r*(*θ*^(*j* − 1)^*∣θ*^(*∗*)^), we accept the generated value and assign *θ*^(*∗*)^ = *θ*^(*j*)^; else, reject the proposal and put *θ*^(*j* − 1)^ = *θ*^(*j*)^.
Step 7: by the same way we will produce and generate *λ*^(*j*)^ by using equation (17) and generate *λ*^(*∗*)^using the normal distribution, and we consider it as the proposal distribution.
Step 8: Make a repetition for the steps from step 4 to step 6 for the parameter *λ* too.
Step 9: Assign *j* = *j* + 1.
Step 10: steps 3 − 9 are repeated for *N* repetitions.
Step 11: We can compute the Bayesian estimate values of the two parameters *θ* and *λ* using SEL function which are, respectively, as below:
  (19) $$\begin{array}{c} {\hat{\theta }}_{BS}=\frac{1}{N-M}\sum_{j=M+1}^{N}\theta^{\left(j\right)}, \end{array}$$
  (20) $$\begin{array}{c} {\hat{\lambda }}_{BS}=\frac{1}{N-M}\sum_{j=M+1}^{N}\lambda^{\left(j\right)}, \end{array}$$
where *M* is the number of iterations that are not considerd in the calculation, and sometime we call it nburn iterations.
Step 12: We can compute the Bayesian estimates values of the two parameters *θ* and *λ* using LINEX loss function which are, respectively, as below:
  (21) $$\begin{array}{c} {\hat{\theta }}_{BL}=\frac{-1}{h}\text{ln}\left[\frac{1}{N-M}\sum_{j=M+1}^{N}e^{-h\theta^{\left(j\right)}}\right], \end{array}$$
  (22) $$\begin{array}{c} {\hat{\lambda }}_{BL}=\frac{-1}{h}\text{ln}\left[\frac{1}{N-M}\sum_{j=M+1}^{N}e^{-h\lambda^{\left(j\right)}}\right]. \end{array}$$

## 5. E-Bayesian Estimation Method

The expectation of Bayesian estimation is referred to as “E-Bayesian estimation” and described as follows.

**Definition 1 .**

Let $\hat{\theta }\left(a,b\right)$ be continuous, then

(23) $$\begin{array}{c} {\hat{\delta }}_{EB}=E\left[\hat{\delta }\left(a,b\right)\right]\\ =\iint_{Q}\hat{\delta }\left(a,b\right)\pi \left(a,b\right)\text{d}a\text{d}b, \end{array}$$

is called the expected Bayesian estimation of *δ* (briefly E-Bayesian estimation) where $\hat{\delta }\left(a,b\right)$ is the Bayesian estimate of *δ* with hyperparameters *a* and *b*, *Q* is the domain of (*a*, *b*), and *π*(*a*, *b*) is the prior PDF of *a* and *b* over *Q*.

From Han [15], the prior parameters (*a*_1_, *a*_2_) and (*b*_1_, *b*_2_) must be picked in order to ensure that *π*_1_(*θ*) and *π*_2_(*λ*)are indeed a pair of declining functions of *θ* and *λ*, respectively. We can get the differentiation of *π*_1_(*θ*) regarding *θ* and *π*_2_(*λ*) with respect to *λ* as the following two equations:

(24) $$\begin{array}{c} \frac{d\pi_{1}\left(\theta \right)}{\text{d}\theta }\propto \theta^{a_{1}-2}\exp\left(-a_{2}\theta \right)\left\{\left(a_{1}-1\right)-a_{2}\theta \right\},\\ \frac{d\pi_{2}\left(\lambda \right)}{\text{d}\lambda }\propto \lambda^{b_{1}-2}\exp\left(-b_{2}\lambda \right)\left\{\left(b_{1}-1\right)-b_{2}\lambda \right\}. \end{array}$$

When 0 < *a*_1_ < 1 and *a*_2_ > 0, (*dπ*_1_(*θ*)/d*θ*) < 0, and when 0 < *b*_1_ < 1 and *b*_2_ > 0, (*dπ*_2_(*λ*)/d*λ*) < 0. Thus, *π*_1_(*θ*) and *π*_2_(*λ*) are decreasing functions for *θ* and *λ*, respectively. We make the assumption that the hyperparameters *a*_*j*_ and *b*_*j*_, *j* = 1,2, are independent and have the bivariate PDF given by

(25) $$\begin{array}{c} \pi \left(a_{1},a_{2}\right)=\pi_{1}\left(a_{1}\right)\pi_{2}\left(a_{2}\right),\\ \pi \left(b_{1},b_{2}\right)=\pi_{1}\left(b_{1}\right)\pi_{2}\left(b_{2}\right). \end{array}$$

In order to get the E-Bayesian estimates of the two parameters *θ* and *λ*, we suggest the prior PDFs of (*a*_1_, *a*_2_) and (*b*_1_, *b*_2_) to clarify the impact of them on the E-Bayesian estimates of *θ* and *λ*. The prior PDFs of (*a*_1_, *a*_2_) and (*b*_1_, *b*_2_) are, respectively, given as follows:

(26) $$\begin{array}{c} \begin{array}{c} \begin{array}{c} \pi_{1}\left(a_{1},a_{2}\right)=\frac{2a_{1}}{c_{1}},\;0<a_{1}<1,0<a_{2}<c_{1},\\ \pi_{2}\left(a_{1},a_{2}\right)=\frac{2a_{2}}{c_{1}^{2}},\;0<a_{1}<1,0<a_{2}<c_{1},\\ \pi_{3}\left(a_{1},a_{2}\right)=\frac{3a_{2}^{2}}{c_{1}^{3}},\;0<a_{1}<1,0<a_{2}<c_{1}, \end{array} \end{array} \end{array}$$

and

(27) $$\begin{array}{c} \begin{array}{c} \begin{array}{c} \pi_{1}\left(b_{1},b_{2}\right)=\frac{2b_{1}}{c_{2}},\;0<b_{1}<1,0<b_{2}<c_{2},\\ \pi_{2}\left(b_{1},b_{2}\right)=\frac{2b_{2}}{c_{2}^{2}},\;0<b_{1}<1,0<b_{2}<c_{2},\\ \pi_{3}\left(b_{1},b_{2}\right)=\frac{3b_{2}^{2}}{c_{2}^{3}},\;0<b_{1}<1,0<b_{2}<c_{2}. \end{array} \end{array} \end{array}$$

### 5.1. E-Bayesian Estimates Based on the Loss Functions

By substituting from (19) and (26) in (23), the E-Bayesian estimate of *θ* using the SEL function can be easily evaluated using the following equation:

(28) $$\begin{array}{c} {\hat{\theta }}_{EB}=E\left[\hat{\theta_{B}}\left(a_{1},a_{2}\right)\right]=\iint_{Q}\hat{\theta_{B}}\left(a_{1},a_{2}\right)\pi_{i}\left(a_{1},a_{2}\right)\text{d}a_{1}\text{d}a_{2},\;i=1,2,3, \end{array}$$

and the E-Bayesian estimate of *λ* based on the SEL function is computed by substituting in (23) from (20) and (27) as follows:

(29) $$\begin{array}{c} {\hat{\lambda }}_{EB}=E\left[{\hat{\lambda }}_{B}\left(b_{1},b_{2}\right)\right]=\iint_{Q}{\hat{\lambda }}_{B}\left(b_{1},b_{2}\right)\pi_{i}\left(b_{1},b_{2}\right)\text{d}b_{1}\text{d}b_{2},\;i=1,2,3, \end{array}$$

where ${\hat{\theta }}_{B}\left(a_{1},a_{2}\right)$ and ${\hat{\lambda }}_{B}\left(b_{1},b_{2}\right)$ are the estimates of the Bayesian method for *θ* and *λ* by applying the SEL function.

Similarly, by substituting from (21) and (26) in (23), we get the E-Bayesian estimate of *θ* based on the LINEX loss function as follows:

(30) $$\begin{array}{c} {\hat{\theta }}_{EB}=E\left[{\hat{\theta }}_{BL}\left(a_{1},a_{2}\right)\right]=\iint_{Q}{\hat{\theta }}_{BL}\left(a_{1},a_{2}\right)\pi_{i}\left(a_{1},a_{2}\right)\text{d}a_{1}\text{d}a_{2},\;i=1,2,3, \end{array}$$

and the E-Bayesian estimate of *λ* based on the LINEX loss function is obtained by substituting in (23) from (22) and (27) as follows:

(31) $$\begin{array}{c} {\hat{\lambda }}_{EB}=E\left[{\hat{\lambda }}_{BL}\left(b_{1},b_{2}\right)\right]=\iint_{Q}{\hat{\lambda }}_{BL}\left(b_{1},b_{2}\right)\pi_{i}\left(b_{1},b_{2}\right)\text{d}b_{1}\text{d}b_{2},\;i=1,2,3, \end{array}$$

where ${\hat{\theta }}_{BL}\left(a_{1},a_{2}\right)$ and ${\hat{\lambda }}_{BL}\left(b_{1},b_{2}\right)$ are the estimates of *θ* and *λ* regarding the Bayesian method under the LINEX loss function. For details, one can see, Han [16]; Jaheen and Okasha [17]; Okasha [18]; Rabie and Li [19]; Rabie and Li [20]; and Rabie [21]. Before progressing, we describe procedures of the simulation study used in this paper.

## 6. Simulation Study

Here, we provide the simulation results according to the following steps:

Specify the values of *n*, *r*_1_, *r*_2_, *k*_1_, *k*_2_, *h*, *a*_1_, *a*_2_, *b*_1_, *b*_2_, *c*_1_, *c*_2_,  and *T*.
First we use the uniform distribution to genrate a random sample *n* from *U*(0,1).
We will choose *n*_1_ items that are selected randomly, from *n* items, and subject them to the normal usage situations.
Compute *n*_2_ = *n* − *n*_1_. These units, allocations, are subjected to stress conditions.
Indicate the initial values used in generating data for the two paramters *θ* and *λ*.
Now we will produce a generalized Type-I hybrid censored sample from the EGD CSPALT model using the inverse function method by solving *U* = (1 − *e*^−*x*^(*x* + 1))^*θ*^ regarding *x* for a typically use situations and by solving *U* = (1 − *e*^−*λy*^(*λy* + 1))^*θ*^ with respect to *y*, for stress use situations.
We build a Markov chain containing 11,000 data by using the Metropolis–Hastings algorithm of *θ* and *λ*, and we will not consider the first 1000 values as they are very affected with the initial values.
${\hat{\theta }}_{BS}$ and ${\hat{\lambda }}_{BS}$ are the Bayesian estimation of the two parameters using the SEL function which can be easily computed by using equation (19) and equation (20), respectively.
${\hat{\theta }}_{BL}$ and ${\hat{\lambda }}_{BL}$ are the E-Bayesian estimation of the two parameters using the LINEX loss function which can be easily computed by using equation (21) and equation (22), respectively.
${\hat{\theta }}_{EBS}$ and ${\hat{\lambda }}_{EBS}$are the E-Bayesian estimation of the two parameters using the SEL function which can be easily computed by using equation (28) and equation (29), respectively.
${\hat{\theta }}_{EBL}$ and ${\hat{\lambda }}_{EBL}$are the E-Bayesian estimation of the two parameters using the LINEX loss function which can be easily computed by using equation (30) and (31), equation respectively.
We can compute the mean squared error (MSE) or the estmiated values of the two parameters *θ* and *λ*, which are, respectively, as follows:
  (32) $$\begin{array}{c} MSE\left(\hat{\theta }\right)=\frac{1}{1000}\sum_{j=1}^{1000}{\left(\hat{\theta_{j}}-\theta \right)}^{2},\\ MSE\left(\hat{\lambda }\right)=\frac{1}{1000}\sum_{j=1}^{1000}{\left(\hat{\lambda_{j}}-\lambda \right)}^{2}, \end{array}$$
where $\hat{\theta }$ is considerd as the estimated values for *θ* and $\hat{\lambda }$ is considerd as the estimated values for *λ*.
The numerical outcomes are obtained using MATHEMATICA 8 functions such as FindRoot, NMaximize, NIntegrate, and RandomReal and displayed in Tables 1 and 2.  Table 1 contains the numerical values of the Bayesian, E-Bayesian estimates, also it contains the MLEs, beside these values there is MSE of the parameter *θ*, using the LINEX and SEL functions.  Table 2 contains the numerical values of the Bayesian, E-Bayesian estimates, also it contains the MLEs, beside these values there is MSE of the parameter *λ* using LINEX and SEL functions. By observing the results in Tables 1 and 2, we may infer that the E-Bayesian estimate outperforms the Bayesian estimator for the SEL and LINEX loss functions for the two parameters *θ* and *λ* as we can easily see it having small values for the MSE. Additionally, when the sample size rises, the MSE of Bayesian and E-Bayesian estimators decreases for a sample of size *n* and the censoring time *T* get increased.

## 7. Example of Real-Life Data

In this section, an example of real-life data is provided to investigate the performance of the proposed methods in the application. These data were used by Singh et al. [22], representing the average monthly rainfall obtained from the Information System for Management of Water Resources from the State of So Paulo, including a period of 56 years from 1947 to 2003. Also, they checked the fitting of the given data set through different method of estimation and stated that EGD gives a good fit for these data. These data contain 56 observations listed as follows: 0.2, 3.5, 2.8, 3.7, 8.7, 6.9, 7.4, 0.8, 4.8, 2.5,2.9, 3.1, 4.0, 5.0, 3.8, 3.5, 5.4, 3.3, 2.9, 1.7, 7.3, 2.9, 4.6,1.1, 1.4, 3.9, 6.2, 4.1, 10.8, 3.8, 7.3, 1.8, 6.7, 3.5, 3.2, 5.2, 2.8, 5.2, 5.4, 2.2, 9.9,2.1, 4.7, 5.5, 2.6, 4.1, 5.4, 5.5, 2.1, 1.9, 8.8, 1.3, 24.1, 5.4, 6.2, 2.9.

We suppose that values of data set represent lifetime of failure observations which follow the EGD. Now we will apply the CSPALT when the sample is genralized Type-I hybrid censoring scheme. Such that *n*_1_ = 26 and *n*_2_ = 30, where the first sample and the second sample were selected randomly from the complete sample of size *n* = 56 units. We desire to obtain *r*_1_ = 21 of failures out of *n*_1_ = 26 units, and *k*_1_ = 15 is a bare minimum number of failures that can be accepted out of *n*_1_ = 26 units. While we desire to obtain *r*_2_ = 25 failures out of *n*_2_ = 30 units, *k*_2_ = 20 represents a minimum number of failures is acceptable from *n*_2_ = 30 units. All estimates of *θ* and *λ* are derived based on the same previous procedures and shown in Tables 3 and 4.  Table 3 gives estimates and MSE for the parameter *θ* of ML, Bayesian, and E-Bayesian estimation methods based on SEL and LINEX loss functions.  Table 4 gives the previous criteria for the acceleration factor *λ*. By observing results listed in Tables 3 and 4 regarding the real data set, one can note that the E-Bayesian method is the best compared with both ML and Bayesian estimation methods because of having less MSE. Moreover, the proposed methods are easily applied to the real data and gave good results.

## 8. Conclusion

In this paper, we studied the exponentiated gamma distribution (EGD) with generalized Type-I hybrid censored data under the constant-stress partially accelerated life test (CSPALT) model. We discussed the Bayesian and E-Bayesian estimation methods, as well as the maximum likelihood method, for the distribution parameter and the acceleration factor. The E-Bayesian and Bayesian estimates are obtained by the SEL and the LINEX loss functions. The MCMC method is used for deriving the Bayesian estimates, and then we computed the E-Bayesian estimates. We provided a real data set to clarify the behavior of the methods in the application.

From the results shown in Tables 1–4, we may conclude that the E-Bayesian estimation approach is superior to both ML and Bayesian estimation methods due to its lower MSE. Also, the E-Bayesian estimation method is easy to be applied and convenient to the application. Additionally, by including additional failure items in the CSPALT model with censoring strategies, adequate information about test units is obtained. Additionally, it is shown that the presented methodologies are simply applicable to the CSPALT model and provide acceptable results.

## Figures and Tables

**Table 1:** Average estimates, MSE of MLEs, Bayesian, and E-Bayesian estimates for *θ* under LINEX and SEL at *θ*=0.7, *λ*=1.2, *a*_1_=*b*_1_=0.7, *a*_2_=1.4, *b*_2_=1.2, *h*=1.5,  and *c*_1_=*c*_2_=2.

(*n*_1_, *n*_2_)	*T*	Criteria	${\hat{\theta }}_{MLE}$	Squared error loss				LINEX loss			
(*r*_1_, *r*_2_)	(*k*_1_, *k*_2_)			${\hat{\theta }}_{BS}$	${\hat{\theta }}_{EBS1}$	${\hat{\theta }}_{EBS2}$	${\hat{\theta }}_{EBS3}$	${\hat{\theta }}_{BL}$	${\hat{\theta }}_{EBL1}$	${\hat{\theta }}_{EBL2}$	${\hat{\theta }}_{EBL3}$
(20,25)	2	Mean	0.753 997	1.143 54	0.672 402	0.576 345	0.518 71	1.129 74	0.667 409	0.572 641	0.515 693
(15, 20)	(10, 15)	MSE	0.042 922	0.210 2	0.005 419	0.018 712	0.035 638	0.195 27	0.005 092	0.019 241	0.036 446

	2.5	Mean	0.772 42	1.060 93	0.623 824	0.534 706	0.581 199	1.058 55	0.622 986	0.534 088	0.574 801
		MSE	0.033 419	0.130 29	0.005 811	0.027 328	0.017 594	0.128 58	0.005 939	0.027 533	0.018 685

(30,40)	2	Mean	0.926 18	1.045 55	0.614 784	0.526 958	0.474 262	1.044 07	0.614 261	0.526 572	0.473 949
(20, 30)	(15, 20)	MSE	0.086 35	0.119 41	0.007 264	0.029 945	0.050 959	0.118 39	0.007 353	0.030 078	0.051 1

	2.5	Mean	0.818 958	1.026 42	0.603 538	0.517 318	0.465 586	1.025 98	0.603 384	0.517 205	0.465 494
		MSE	0.019 73	0.106 56	0.009 306	0.033 373	0.054 95	0.106 27	0.009 336	0.033 415	0.054 993

(40,50)	2	Mean	0.885 694	1.035 89	0.609 101	0.522 087	0.469 878	1.034 77	0.608 703	0.521 792	0.469 639
(30, 40)	(20, 30)	MSE	0.060 416	0.113 04	0.008 338	0.031 708	0.053 001	0.112 26	0.008 405	0.031 81	0.053 109

	2.5	Mean	0.857 984	1.010 3	0.594 057	0.509 192	0.458 273	1.010 23	0.594 031	0.509 172	0.458 257
		MSE	0.060 304	0.096 29	0.011 225	0.036 408	0.058 433	0.096 24	0.011 23	0.036 416	0.058 44

**Table 2:** Average estimates, MSE of MLEs, Bayesian, and E-Bayesian estimates for *λ* under LINEX and SEL at *θ*=0.7, *λ*=1.2, *a*_1_=*b*_1_=0.7, *a*_2_=1.4, *b*_2_=1.2, *h*=1.5,  and *c*_1_=*c*_2_=2.

(*n*_1_, *n*_2_)	*T*	Criteria	${\hat{\lambda }}_{MLE}$	Squared error loss				LINEX loss			
(*r*_1_, *r*_2_)	(*k*_1_, *k*_2_)			${\hat{\lambda }}_{BS}$	${\hat{\lambda }}_{EBS1}$	${\hat{\lambda }}_{EBS2}$	${\hat{\lambda }}_{EBS3}$	${\hat{\lambda }}_{BL}$	${\hat{\lambda }}_{EBL1}$	${\hat{\lambda }}_{EBL2}$	${\hat{\lambda }}_{EBL3}$
(20,25)	2	Mean	1.011 88	1.559 98	1.070 15	1.070 15	1.123 65	1.525 09	1.053 51	1.053 51	1.105 34
(15, 20)	(10, 15)	MSE	0.123 564	0.181 31	0.041 203	0.041 203	0.032 665	0.151 84	0.043 967	0.043 967	0.033 679

	2.5	Mean	1.002 44	1.555 65	1.067 17	1.067 17	1.156 64	1.520 02	1.050 2	1.050 2	1.137 03
		MSE	0.051 584	0.133 96	0.021 163	0.021 163	0.015 552	0.108 92	0.025 639	0.025 639	0.016 705

(30,40)	2	Mean	1.126 31	1.691 17	1.160 14	1.160 14	1.218 15	1.663 99	1.147 25	1.147 25	1.203 95
(20, 30)	(15, 20)	MSE	0.044 112	0.244 34	0.003 043	0.003 043	0.001 933	0.218 26	0.004 199	0.004 199	0.001 575

	2.5	Mean	0.949 765	1.511 48	1.036 87	1.036 87	1.088 72	1.490 4	1.026 89	1.026 89	1.077 72
		MSE	0.062 93	0.104 28	0.030 028	0.030 028	0.016 152	0.091 08	0.033 216	0.033 216	0.018 525

(40,50)	2	Mean	1.087 32	1.552 71	1.065 16	1.065 16	1.118 42	1.534 22	1.056 4	1.056 4	1.108 77
(30, 40)	(20, 30)	MSE	0.045 626	0.133 46	0.022 442	0.022 442	0.011 353	0.120 31	0.024 734	0.024 734	0.012 851

	2.5	Mean	1.034 61	1.469 1	1.007 8	1.007 8	1.058 19	1.452 11	0.999 774	0.999 774	1.049 35
		MSE	0.038 813	0.075 08	0.038 195	0.038 195	0.021 492	0.066 12	0.041 312	0.041 312	0.024 042

**Table 3:** Real data set: average estimates, MSE of MLEs, Bayesian, and E-Bayesian estimates for *θ* under LINEX and SEL functions when *θ*=2, *λ*=1.24, *a*_1_=0.8, *b*_1_=0.7, *a*_2_=1.6, *b*_2_=1.4, *h*=1.5,  and *c*_1_=*c*_2_=2.

(*n*_1_, *n*_2_)	*T*	Criteria	${\hat{\theta }}_{MLE}$	Squared error loss				LINEX loss			
(*r*_1_, *r*_2_)	(*k*_1_, *k*_2_)			${\hat{\theta }}_{BS}$	${\hat{\theta }}_{EBS1}$	${\hat{\theta }}_{EBS2}$	${\hat{\theta }}_{EBS3}$	${\hat{\theta }}_{BL}$	${\hat{\theta }}_{EBL1}$	${\hat{\theta }}_{EBL2}$	${\hat{\theta }}_{EBL3}$
(26,30)	4.8	Mean	2.567 4	2.672 82	1.833 55	1.833 55	1.925 23	2.624 82	1.811 65	1.811 65	1.901
(21, 25)	(15, 20)	MSE	0.321 945	0.452 72	0.027 722	0.027 722	0.005 609	0.390 45	0.035 495	0.035 495	0.009 824
(26,30)	5.8	Mean	2.578 21	2.679 14	1.837 89	1.837 89	1.929 78	2.632 4	1.816 58	1.816 58	1.906 21
(21, 25)	(15, 20)	MSE	0.334 329	0.461 27	0.026 299	0.026 299	0.004 951	0.399 98	0.033 665	0.033 665	0.008 821

**Table 4:** Real data set: average estimates, MSE of MLEs, Bayesian, and E-Bayesian estimates for *λ* under LINEX and SEL functions when *θ*=2, *λ*=1.24, *a*_1_=0.8, *b*_1_=0.7, *a*_2_=1.6, *b*_2_=1.4, *h*=1.5,  and *c*_1_=*c*_2_=2.

(*n*_1_, *n*_2_)	*T*	Criteria	${\hat{\lambda }}_{MLE}$	Squared error loss				LINEX loss			
(*r*_1_, *r*_2_)	(*k*_1_, *k*_2_)			${\hat{\lambda }}_{BS}$	${\hat{\lambda }}_{EBS1}$	${\hat{\lambda }}_{EBS2}$	${\hat{\lambda }}_{EBS3}$	${\hat{\lambda }}_{BL}$	${\hat{\lambda }}_{EBL1}$	${\hat{\lambda }}_{EBL2}$	${\hat{\lambda }}_{EBL3}$
(26,30)	4.8	Mean	1.027 72	1.062 14	1.087 63	1.087 63	1.305 15	1.059 94	1.085 33	1.085 33	1.301 87
(21, 25)	(15, 20)	MSE	0.045 063	0.031 64	0.023 226	0.023 226	0.023 226	0.004 258	0.023 932	0.023 932	0.003 84
(26,30)	5.8	Mean	1.029 65	1.062 48	1.087 98	1.087 98	1.305 58	1.060 27	1.085 66	1.085 66	1.302 27
(21, 25)	(15, 20)	MSE	0.044 246	0.031 52	0.023 12	0.023 12	0.004 316	0.032 31	0.023 829	0.023 829	0.003 891

## Data Availability

The data used to support this study are included within the article.
